# Supplementary material for: Structure of the exportin Xpo4 in complex with RanGTP and the hypusine-containing translation factor eIF5A
Source: Nat Commun. 2016 Jun 16;7:11952. doi: 10.1038/ncomms11952 (PMC4912631; doi:10.1038/ncomms11952)
Supplement: Supplementary Information — Supplementary Figures 1-5 and Supplementary Reference. [file ncomms11952-s1.pdf]

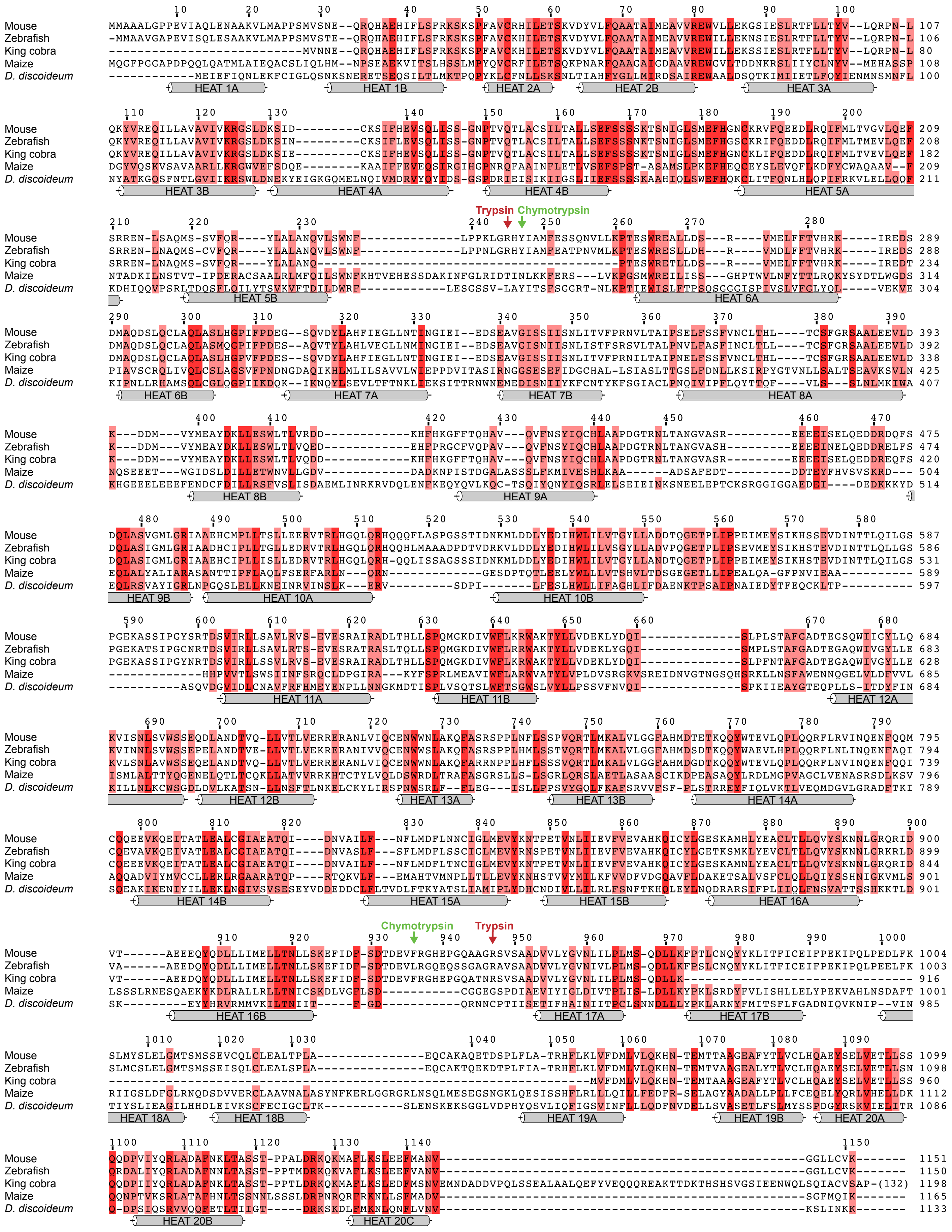
Mouse
Zebrafish
King cobra
Maize
D. discoideum
HEAT 1A
HEAT 1B
HEAT 2A
HEAT 2B
HEAT 3A
HEAT 3B
HEAT 4A
HEAT 4B
HEAT 5A
Trypsin
Chymotrypsin
HEAT 5B
HEAT 6A
HEAT 6B
HEAT 7A
HEAT 7B
HEAT 8A
HEAT 8B
HEAT 9A
HEAT 9B
HEAT 10A
HEAT 10B
HEAT 11A
HEAT 11B
HEAT 12A
HEAT 12B
HEAT 13A
HEAT 13B
HEAT 14A
HEAT 14B
HEAT 15A
HEAT 15B
HEAT 16A
Chymotrypsin
Trypsin
HEAT 16B
HEAT 17A
HEAT 17B
HEAT 18A
HEAT 18B
HEAT 19A
HEAT 19B
HEAT 20A
HEAT 20B
HEAT 20C

Supplementary Figure 1 Sequence alignment of Xpo4 orthologues.

The alignment includes Xpo4 from mouse *(M. musculus*), zebrafish (*D. rerio*), king cobra (*O. hannah*), maize (*Z. mays*), and *D. discoideum*. Absolutely conserved residues are highlighted in dark red boxes, residues conserved in four out of five sequences in light red boxes. Identified protease cleavage sites are marked with arrows and indicated accordingly. The positions of the HEAT repeat helices of mouse Xpo4 are labeled and positioned below the alignment.

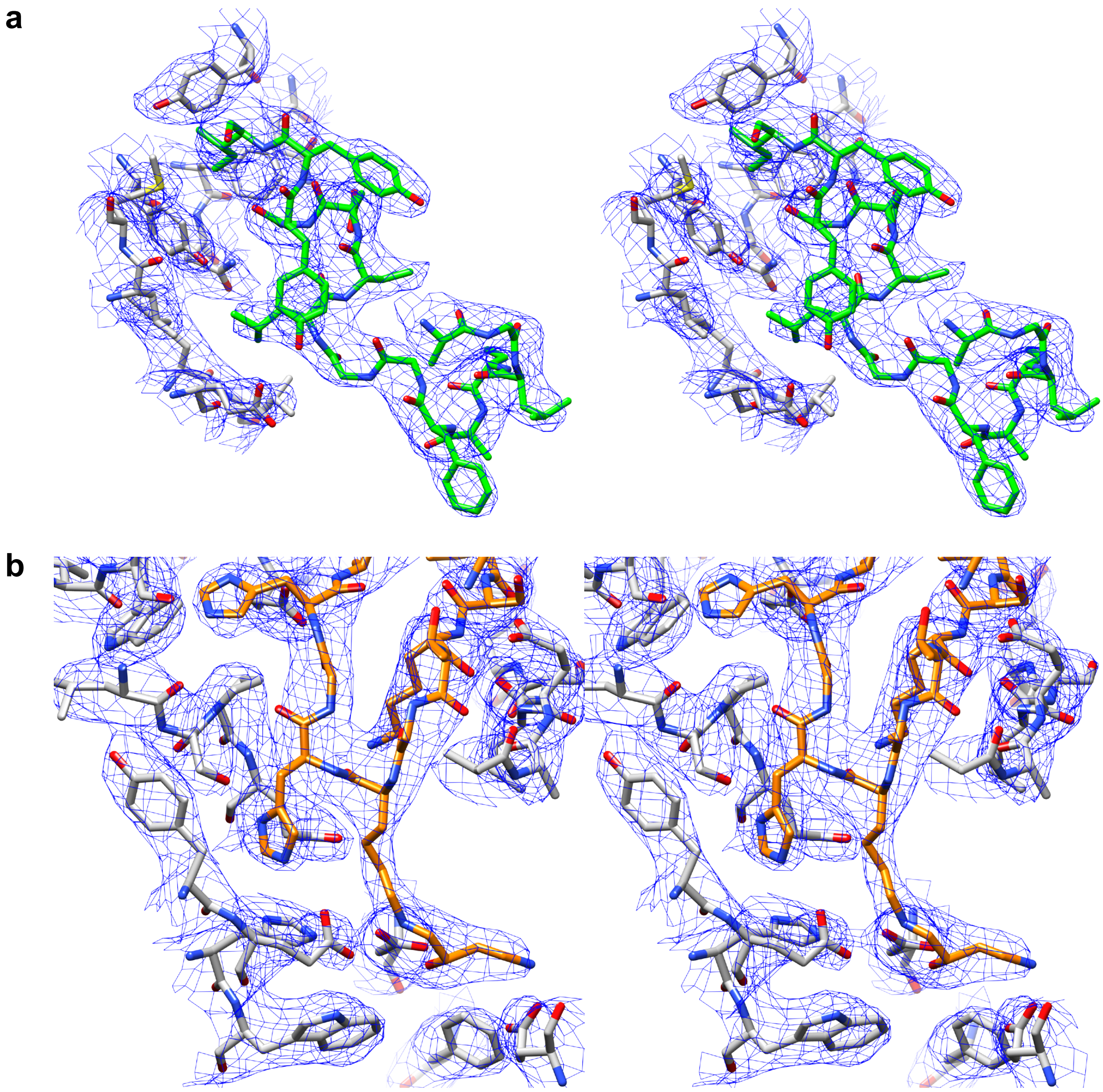

Supplementary Figure 2 Stereo views of the electron density of the export complex structure.

The electron density of the refined 2Fo-Fc map (contoured at 1.0 σ) is shown as blue mesh, with the stick representation of the final model superposed.

(**a**) Stereo view of the switch II region of Ran (carbon atoms in green) interacting with Xpo4 (carbon atoms in grey).

(**b**) Stereo view showing the hypusine-containing loop of eIF5A (carbon atoms in orange) docking into the acidic pocket of Xpo4.

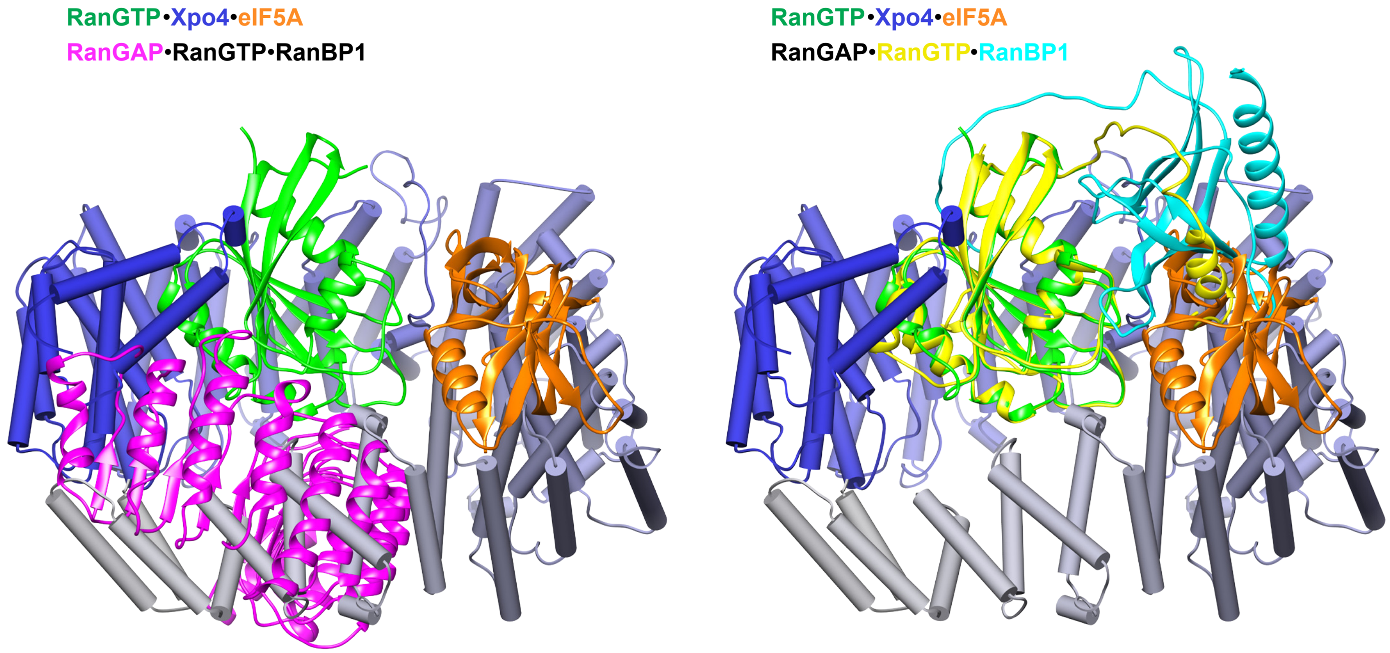

Supplementary Figure 3 Cytoplasmic disassembly of the export complex.

RanGTP•Xpo4•eIF5A complex is overlaid with the RanGAP•RanGTP•RanBP1 structure (PDB ID 1K5D; ref. 1). The structures are aligned via Ran. The eIF5A export complex is shown in a similar color-coding and orientation as in Figure 3 (middle panel). On the left, RanGAP (magenta) is shown in a ribbon representation. On the right, RanGTP is shown in yellow and RanBP1 in cyan. Note the severe clashes of RanGAP with the C-terminus of Xpo4 and of RanGTP•RanBP1 with Xpo4 and eIF5A.

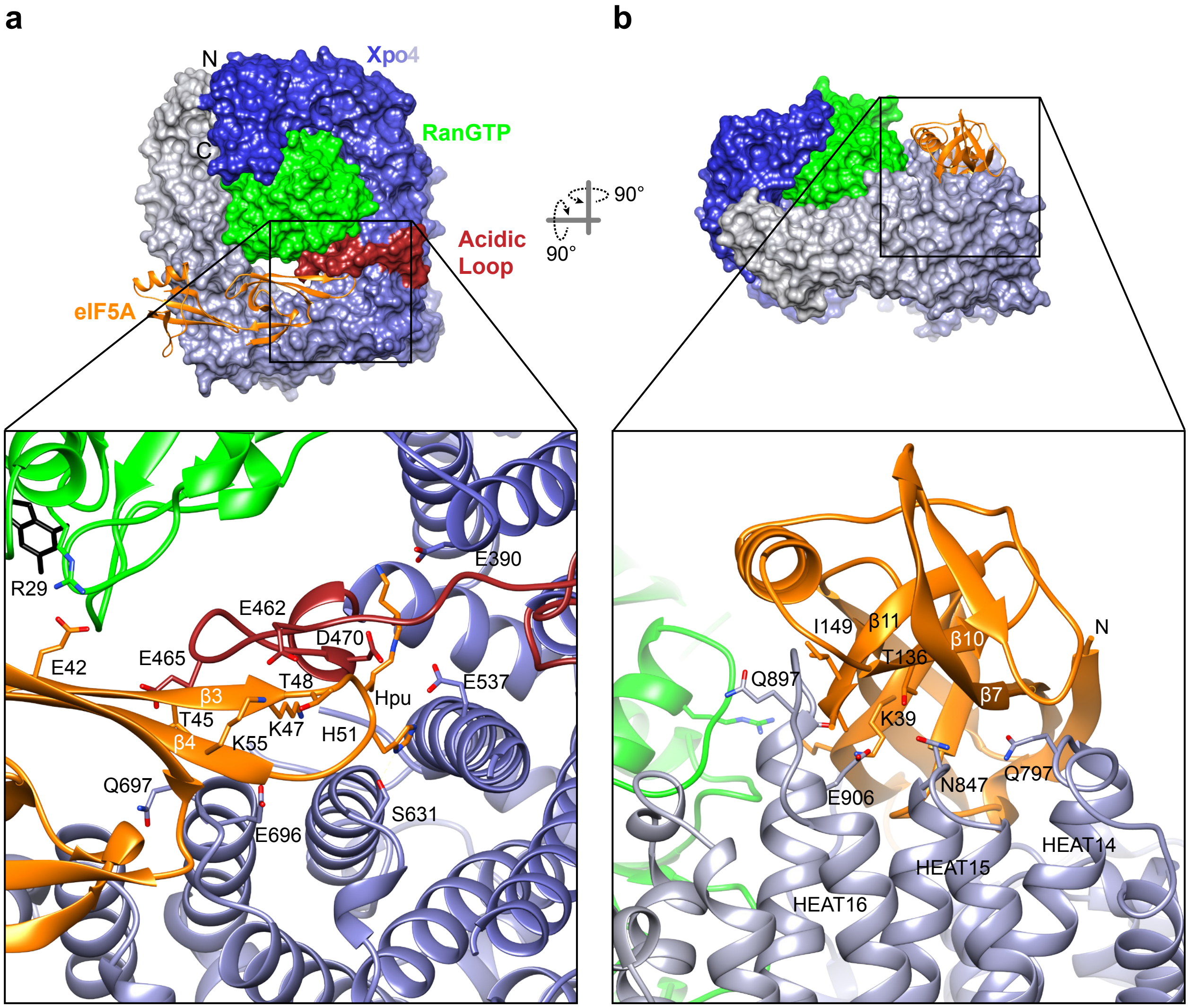

Supplementary Figure 4 Detailed interactions of eIF5A with RanGTP•Xpo4.

Docking of the N-terminal (**a**) and C-terminal (**b**) domains of eIF5A on RanGTP•Xpo4. Xpo4 and Ran are depicted as surface, whereas eIF5A is shown in a ribbon (upper panels). The magnified views (bottom panels) show the interacting residues.

Fig. 5a

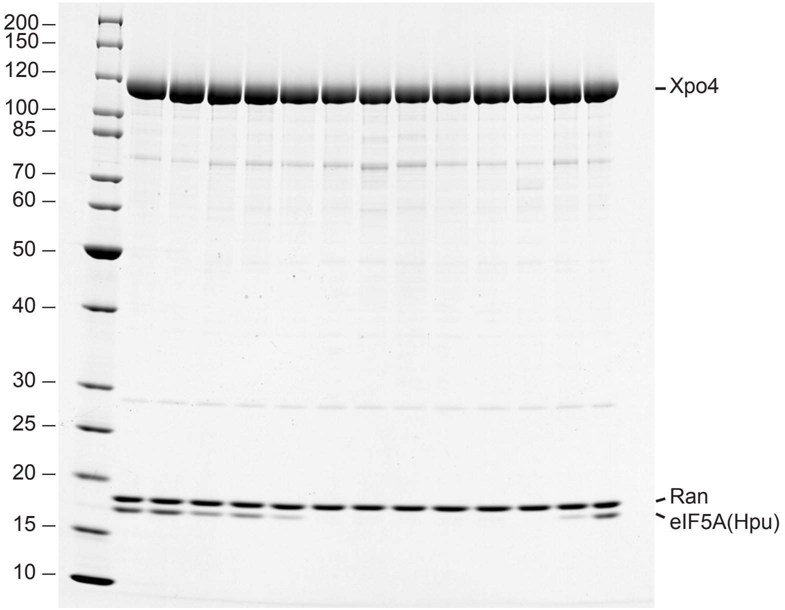

Fig. 5c

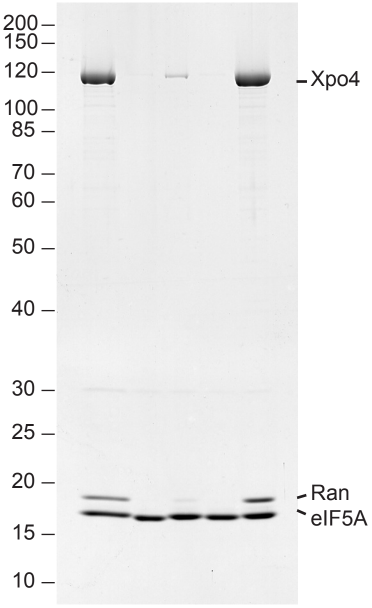

Supplementary Figure 5 The original scans of the gels used in Figure 7

Supplementary Reference

1. Seewald, M. J., Korner, C., Wittinghofer, A. & Vetter, I. R. RanGAP mediates GTP hydrolysis without an arginine finger. *Nature* **415**, 662-666 (2002).
